# Improving Quality of Patient Data for Treatment of Multidrug- or Rifampin-Resistant Tuberculosis

**DOI:** 10.3201/eid2603.190997

**Published:** 2020-03

**Authors:** Jonathon R. Campbell, Dennis Falzon, Fuad Mirzayev, Ernesto Jaramillo, Giovanni Battista Migliori, Carole D. Mitnick, Norbert Ndjeka, Dick Menzies

**Affiliations:** McGill University, Montreal, Québec, Canada (J.R. Campbell, D. Menzies);; Research Institute of the McGill University Health Centre, Montreal (J.R. Campbell, D. Menzies);; World Health Organization, Geneva, Switzerland (D. Falzon, F. Mirzayev, E. Jaramillo);; World Health Organization Collaborating Centre for Tuberculosis and Lung Diseases, Tradate, Italy (G.B. Migliori);; Harvard Medical School, Boston, Massachusetts, USA (C.D. Mitnick);; South African National Department of Health, Pretoria, South Africa (N. Ndjeka); Montréal Chest Institute, Montreal (D. Menzies)

**Keywords:** tuberculosis, multidrug resistance, therapy data collection, data collection standards, meta-analysis, evidence-based medicine, statistics and numerical data, practice guideline, antimicrobial resistance, bacteria, MDR TB, RR-TB, TB

## Abstract

International policy for treatment of multidrug- and rifampin-resistant tuberculosis (MDR/RR TB) relies largely on individual patient data (IPD) from observational studies of patients treated under routine conditions. We prepared guidance on which data to collect and what measures could improve consistency and utility for future evidence-based recommendations. We highlight critical stages in data collection at which improvements to uniformity, accuracy, and completeness could add value to IPD quality. Through a repetitive development process, we suggest essential patient- and treatment-related characteristics that should be collected by prospective contributors of observational IPD in MDR/RR TB.

The treatment of multidrug- and rifampin-resistant tuberculosis (MDR/RR TB) is complex. Treatment requires a combination of multiple agents and often needs to be individualized, taking numerous considerations into account (*1*,*2*). Patients may have different concurrent conditions, such as HIV infection or diabetes; furthermore, the disease may vary in terms of extent, both in the lungs themselves (i.e., through presence of lung cavitation, bilateral disease, or both) and in other extrapulmonary sites (*2*). The pattern of additional resistances to other key agents used in second-line TB regimens may differ, depending on previous treatment received by the individual patient (either first-line or second-line medicines) and the epidemiologic setting (*3*,*4*). In different centers, the protocol for microbiologic monitoring may vary from none to monthly sputum smear microscopy and cultures with periodic drug-susceptibility testing during treatment, which, at times, continues after successful treatment to detect recurrence (*2*). The treatment given may be affected by the experience and expertise of the healthcare providers, as well as the cost of medicines and their availability (*5*). The use of adjunct therapies such as surgery (*6*), hospitalization, and patient support for treatment adherence (*7*), such as patient-centered directly observed therapy (DOT), also varies by program. The occurrence of adverse drug reactions to second-line TB drugs is common (*1*,*8*) and may be managed differently in different settings, particularly the permanent withdrawal of certain agents. All these factors result in wide variation in patient management and outcomes.

There is a shortage of high-quality randomized controlled trial (RCT) data for MDR/RR TB drugs (*9*), and currently available evidence is not adequately powered for patient outcomes *(**10**–**14*). Although several notable RCTs evaluating standardized treatments are in the pipeline (*15*), no single regimen is likely to address the entire spectrum of clinical features that patients with MDR/RR TB have. This disease will largely require different treatment approaches individualized to the specific characteristics of the patient and the drug susceptibility profile of the strain.

Until the results of RCTs become available, new evidence for treatment of MDR/RR TB must be derived largely from observational studies. More than 150,000 MDR/RR TB patients initiate therapy each year worldwide, representing a wealth of potential data (*16*). These patients have an enormous diversity of clinical characteristics, many (e.g., pregnant women) are underrepresented in RCTs, and they are treated with widely varying regimens within health systems with different resources and capacities (*17*). This reflects the various scenarios in which global recommendations made by the World Health Organization (WHO) are expected to be applied and thus observational data can play a critical role in recommendation development.

Still, potential problems exist with use of observational data. The greatest are the potential for different forms of confounding and bias (*18*,*19*). This can be mitigated, at least partially, by careful adjustment for the many potential confounding factors, including age, prior treatment history, extent of drug resistance and disease, concurrent conditions, and treatment response (*2*). Adequate adjustment for confounders necessitates that information is accurately recorded for all patients treated, which is often not the case; missing data represents a second major potential limitation of observational data. Certain information may be missing for all patients in some centers, which could be the result of lack of capacity (e.g., radiography findings are missing because chest radiographs are not accessible) or the required information never being gathered or reported. Alternatively, other key data on determinants of patient outcomes, such as frequency and timing of regimen change, may be variably collected across studies. This may be caused by differences in the monitoring schedules, the data collection systems, and the medications used between studies and over time. At times, data collection may be directly related to determinants of outcome (e.g., length of QT-interval is more carefully measured and recorded in patients with multiple risks for cardiotoxicity) and can lead to measurement or ascertainment biases that are difficult to detect or mitigate appropriately.

Despite these problems, observational data have been collected from many centers and pooled together, allowing for individual patient data meta-analyses (IPD-MAs). Since 2010, when WHO and other organizations started using GRADE for drug-resistant TB treatment guidelines (*20*), WHO recommendations on the type, composition, and duration of second-line TB regimens have been based largely on evidence from observational studies of patients treated under field conditions (*21**–**25*). Ahead of the WHO MDR/RR TB guideline update in 2018, a public call was made for contributors to report IPD conforming to certain criteria and a specific data dictionary (*26*). This cal lpermitted the inclusion of more recent programmatic data that may have never been published, increasing the breadth and relevance of the information available for study.

Overall, well-gathered, carefully documented, and complete observational datasets represent a valuable resource for assessing treatment regimens in MDR/RR TB. If efforts are made to safeguard the uniformity and quality of these data in terms of accuracy, consistency, and completeness, it is possible to accrue sufficient information for large numbers patients treated for MDR/RR TB each year, and to generate evidence within 1–2 years to address critical questions, such as the optimal duration of the newly recommended all-oral MDR/RR TB regimen and the safety profile of new drugs (*1*). In response to our experiences with IPD management and analysis, most recently to update the WHO MDR/RR TB treatment recommendations in 2018, and recognizing the urgent need for guidance, this article highlights how to improve the quality and completeness of future IPD for MDR/RR TB and provide guidance for researchers in other disease areas facing similar problems (*27**–**30*).

## Aim and Scope of Guidance

Improving the completeness and quality of routinely collected data represents a relatively small marginal cost after all other expenditures incurred during care of patients with MDR/RR TB (*31*). Consolidating routinely reported data into high quality observational datasets and pooling these to perform multicentric IPD-MAs is a very attractive option to inform future MDR/RR TB treatment guidelines in the coming years, building on a proven track record (*2*,*21**–**23*,*32**–**34*).

The content of this guidance is meant for coordinators of MDR/RR TB treatment who intend to share their experience in patient care to the benefit of national and global treatment policy following several data-sharing principles (Table 1). This guidance is intended to instruct potential contributors on the utility of their potential observational IPD and aid them in subscribing to key quality and completeness measures to create a database with high quality IPD composed of key variables on patient demographics, clinical characteristics, treatment details and covariates, as well as treatment outcomes in MDR/RR TB patients, and contributing the IPD to a pooled data repository that can be shared internationally to allow for analysis that will inform future evidence-based treatment guidelines.

**Table 1:** Data-sharing principles for contributors of individual patient data for multidrug-/rifampin-resistant tuberculosis

Principle	Additional notes
Data contributed to the IPD should be coded to remove identifying information.	• All names, the date of birth, address, telephone number, and other easily identifying personal information must be removed (e.g., national identification or health insurance numbers). • Each participant contributed should be re-coded with a new IPD identification number that is mapped to the original identification number retained by the contributing investigator, group, and/or program. • Dates of events (e.g., treatment start, cultures, medication changes) should be retained in the sent participant data file. • Other local rules for encoding and other data protection measures should be followed.
The contributing investigator, group, and/or program retains ownership of the data and should have permission to share them.	• The transfer of data for use in guideline development or other projects does not constitute transfer of ownership. • Data contributors are free to withdraw their data at any time. • Data must be contributed only if they are permitted by programs or donor agencies. • A data sharing agreement will specify the details of the transfer of data (an example of a “starting point” for these data sharing agreements is contained in the Appendix).
All transfers of data must clear ethics review	• The institutional review board responsible for the bioethics of each contributed dataset should approve that the data can be shared. • All anticipated uses of the data should be reviewed and approved by the institutional review board.
All uses of data are subject to oversight by the collaborative group.	• Ideally 1 individual is designated to liaise with the rest of the contributors of IPD to approve or deny use of their data for current or future analyses and be part of the oversight committee. • The oversight committee reviews proposals for data use and sharing of data.
All data are held centrally in a secure data repository.	• The IPD used for the development of MDR/RR-TB treatment guidelines for the WHO and other entities has been held securely by the McGill University Health Centre (MUHC) under Dick Menzies since 2010. • The MUHC (now a WHO collaborating center) is expected to retain these responsibilities, pending approval of the oversight committee. • Use of data held in this repository follows these principles, with bioethics approval and conforming to the current data sharing agreements signed.

The guidance in this article was developed by 3 staff members of the WHO Global TB Programme (D.F., E.J., F.M.) involved in numerous iterations of the WHO MDR/RR TB guidelines and 5 methodologists, TB clinicians, and evidence reviewers (J.R.C., G.B.M., C.D.M., N.N., D.M.) involved in these and other guidelines. Four cycles of revisions took place, with successive discussions on key variables to collect, standardization of variable collection, and practical measures to suggest for completeness and quality. Although no one else was involved in the writing of the guidance, we acknowledge that we have benefited from the contribution and collective experience of many data contributors who provided data in the past and are acknowledged in publications of IPD-MA (*2*,*21**–**23*,*26*,*32**–**34*).

## The Requirements for Observational Data

Several requirements exist to contributions of patient data. The first requirement is that the data are collected at centers that have the capacity to adequately gather the key information on all patients treated for MDR/RR TB. Centers should also have access to quality-assured medications in sufficient variety that they can treat patients with different drug susceptibility patterns. The centers should have adequate laboratory facilities to enable repeated microbiological testing throughout treatment, including initial and repeated drug susceptibility testing (DST) for all second-line TB medicines used at that center. Centers should develop internal quality assurance protocols and participate in external laboratory assessment programs to uphold the validity of their laboratory testing (*35*,*36*). These measures limit spurious conclusions being drawn about the influence of a medicine on outcomes resulting from exposure to ineffectual medication. The second requirement is that the program treats a relatively large number of patients with diverse demographic, clinical, and treatment characteristics. This is to avoid having patient series that are extreme outliers to the usual practice in a given setting. Nationwide representativeness is not to be expected, but reports of small patient series (e.g., <25) may be extreme outliers and may present challenges to pooling with other records for IPD-MA. However, we encourage reports of any size on subpopulations with limited available data, such as persons with extrapulmonary MDR/RR TB, pregnant women, children, and vulnerable populations. Finally, the center must adopt a quality-assured methodology for the study parameters and organization of data and respect ethical norms and standards for data collection, management, and use of data for research. This necessitates that the clinical data be entered in electronic format. Infrastructure must be in place to support electronic data collection, and personnel who are motivated and trained in data collection must be available. When possible, crosschecks should be performed between this electronic system and national vital statistics and laboratory registries, which provides information on long-term patient disposition.

## Data Capture: Ensuring Accuracy and Completeness

Several practical measures should be undertaken to ensure that data are captured optimally. Upstream of the collection of data, efforts should be made to ensure the quality of these data, including quality assurance of diagnostic work and verification of patient demographic and clinical information with medical histories.

Transcription of data between systems (e.g., from a paper treatment card to an electronic database) is an eminent source of error. Many settings now have the capability to create an electronic medical record at the first encounter with the patient and access it again to prospectively update the details, either at subsequent patient visits or directly from the laboratory. The widespread availability of internet and desktop computers, laptops, tablets, or smartphones makes this feasible in many settings. This practice would have the advantages of improved completeness of patient files and avoidance of transcription and recall errors when compared with other retrospective practices in data collection, such as periodic transfer of data from a paper treatment record during treatment, or after the treatment episode is completed. Within the electronic record system, anonymization procedures to limit the accidental disclosure of sensitive data are necessary. Various quality control measures can be built in to alert the user when implausible, inconsistent, or “out-of-range”/nonstandardized values are entered, or if data are missing, prompting checks and corrections as necessary (Table 2). Finally, the database architecture of the health information system needs to allow for information from patient follow-up encounters to link up seamlessly to those of the initial record of the patient. A unique key in an electronic dataset limits the risk of duplicate records and avoids the need to re-enter identifiers of the patient and health center at each review. Many different packages have been successfully employed for this purpose, including open-source packages that bear no license fees for use and allow customization (*37*).

**Table 2:** Suggested steps to improve the accuracy and completeness of observational IPD

Suggested steps	Additional notes
Persons responsible for capture and entry of data into electronic databases should be appropriately trained.	• This includes obtaining a certificate in good clinical practice and training around the importance of confidentiality. • This also includes training on the basics of MDR/RR-TB, relevant national guidelines, what to collect, how to collect it, and the importance of accuracy in the capture of data. • These principles can be reinforced with detailed guidance for data capture and the definitions of the variables collected at the point of capture (e.g., within the electronic system or within a document kept where data are captured).
Quality control measures (e.g., data safeguards) should be implemented to prevent implausible or “out-of-range” entries.	• A warning can be implemented for continuous variables falling outside plausible ranges (e.g., age outside 0–99 y). • Drop-down lists can be created to reduce/remove need for free form data entry (e.g., including the most common extrapulmonary TB sites within the dropdown or limiting responses for HIV-coinfection status to positive, negative, or not tested). • Safeguards can be logical, which prevent certain data from being entered without a specific response in another section (e.g., CD4 and viral load cannot be filled in unless HIV-coinfection status is positive).
Supervisors should have a standard quality assurance routine (e.g., perform routine follow-up for data accuracy of collected information).	• Supervisors should have simple algorithms developed to detect implausible information that defy inbuilt measures (e.g., patients reported to be receiving a medicine to which their DST shows resistance). • Complete checks should be run on at least 10% of records independently via dual extraction. These checks should be performed regularly and assessed by a supervisor with the goal of 95% accuracy. • Corrective steps should be taken (e.g., further training, more comprehensive or routine checks of variables) when accuracy of data collection is an issue.
Concurrent checks for data completeness should be performed with assessments of accuracy.	• Reminders can be developed that automatically signal that certain variables are not completed each time a patient record is updated. • In addition, preventing the “finalization” of a patient file until all variables are entered can be implemented—however, files should still be permitted to be saved, and other files opened and populated while patient files await finalization. • Completeness of data is of utmost importance—high frequency of absence of certain information may necessitate exclusion of entire datasets from particular analyses for which these data are required.

## Description of Data Elements

This section highlights key items to capture within an electronic register (or database) for use in national or global analyses. The electronic medical record may contain other valuable information for programmatic management and policy making, such as health-related quality of life measurements, which may be of interest to programs, but which have not traditionally been used in analyses to date. The variables to be collected are those that are necessary to assess exposure (e.g., drugs, duration), potential confounders (e.g., concurrent conditions, resistance), response to treatment (e.g., microbiology, molecular biology, clinical signs and symptoms, and radiograph results), and adverse events (AEs). They also need to gather information which will be used to adjust observed effects by patient strata (e.g., by age, previous treatment history, or disease extent). A data dictionary defining variables and their preferred coding format is contained in the Appendix; the most up-to-date version of this data dictionary and accompanying explanations are held at https://www.mcgill.ca/tb). This list of variables is what is optimally preferred and what contributors should strive for, however if certain data elements are missing from a patient series, the records may still be useful for specific analyses of safety or effectiveness. Further included in the Appendix are standard abbreviations for TB and antiretroviral drugs, standard system organ classes for AEs (*38*), and standardized definitions for patient outcomes (*39*,*40*). We discuss variables that require further elaboration in the subsequent sections.

## Initial (Baseline/Pretreatment)

Several baseline/pretreatment factors exist that affect the prognosis of patients with MDR/RR TB. Apart from typical demographic characteristics, complete collection of information on patients’ habits and concurrent conditions is essential, as the true effect that many of these factors have on treatment outcomes is uncertain. Collection of CD4 counts, viral load, and antiretroviral therapy regimens in HIV-infected persons is essential; additional information on hepatitis B/C status, diabetes mellitus, and mental health disorders may also be useful. Although universally accepted definitions for smoking exist (*41*), this is not the case for alcohol consumption; contributors are encouraged to closely collect the alcohol-related variables in the data dictionary. The occurrence of cavitation and bilateral pulmonary disease is key to a better understanding of their effect on patient outcomes and to the classification of extent of disease. However, recording of radiologic findings in pulmonary MDR/RR TB is not standardized between reporting center and at times are missing. For microbiologic and DST results, several factors may compromise a program’s ability to collect a sample exactly at treatment start. We suggest that baseline tests should be included only if they are performed on samples collected within 3 months before, or 1 month after, start of treatment. DST results should be reported for rifampin and for every medicine used in the regimen for which a WHO-approved laboratory method exists.

## Treatment and Follow-Up Information

All measures that are repeated throughout treatment to inform treatment decisions and those that could affect treatment outcomes should be collected. It is perhaps most crucial to completely and accurately collect information regarding treatment type, duration, and composition. According to current standards, shorter MDR/RR TB regimens are those intended to last for <12 months, whereas longer regimens are intended to last for >18 months (*1*). Details for patients who had to transition from shorter to longer regimens must be reported. For each drug used in the regimen, ideally the day the drug was introduced into the regimen and the day the drug was permanently withdrawn (e.g., because of provider or patient decision or an adverse event) should be recorded. In programs in which this is not possible, new data elements can be added to the dictionary that would capture the patient’s regimen every 1–2 months, using standard abbreviations (Appendix). Adherence support, either in the form of in-person observation or with digital tools, is a common component of MDR/RR TB treatment. Data should be collected regarding its use and frequency. The data dictionary contains variables to record monthly follow-up sputum samples for smear microscopy and culture, with collection of culture results prioritized (*1*,*42*). Programs may also opt to simply report the date when each sputum sample was taken and the accompanying smear and culture result. Regardless of reporting choice, all results obtained should be recorded. Reporting of repeated DST is essential to detect acquired resistances; changes in the resistance patterns must be reported. Only thoracic surgery performed as an adjunctive therapy for MDR/RR TB should be reported.

The reporting of AEs in TB patients is highly valuable, but is often difficult to standardize. AEs of mild and moderate severity are very frequent in patients on TB treatment (*1*,*8*); including all of them in the IPD would be excessive. The AEs that should be entered and reported are drug-related AEs that are considered serious (*43*) or cases in which an agent is stopped for >48 hours by the provider because of a suspected or confirmed drug-related AE. In addition, information about whether the suspected or responsible agent is subsequently stopped permanently should be provided. Data in the “adverse event information” section should also be completed in the case of death that is suspected or confirmed to be drug-related. Characteristics of the AE that should be reported include the system or organ class affected, the agent(s) considered responsible, the severity, and the outcome. The severity should be graded using international standards, such as those of the National Cancer Institute (*44*) or other recommended scales (*43*,*45*). Centers may develop their own resources for the investigation and management of common AEs (e.g., by adapting the contents of manuals [*46*]).

## Treatment Outcomes

End-of-treatment outcomes must be specified according to WHO standards to ensure uniformity. The set of definitions used must be specified, with preference currently given to 2013 criteria (*1*). Ideally, endpoint assignment would be systematically verified. Culture conversion (defined as the date of the first negative culture, when >2 consecutive cultures, >28 days apart, are negative) and culture reversion (defined as the date of the first positive culture, when >2 consecutive cultures, >28 days apart, are positive after culture conversion) should be reported (*1*). Recurrence (because of true relapse or reinfection) information is valuable but scarce and difficult to collect because it requires follow-up after completion of treatment. The possibility to distinguish true relapses from a new infection among recurrences requires genotyping or sequencing that, to date, is done only in specialized laboratories, limiting its use in routine care (*47*). Monitoring patients for >12 months after successful completion of treatment would provide valuable information. If recurrence is monitored and reported, the exact duration of follow-up must be specified.

## Discussion

We present a framework for observational data collection outlining key variables to collect to ensure uniformity in global MDR/RR TB patient data and provide practical measures to be taken to ensure data quality and completeness. National or regional TB programs, as well as operational research projects, patient series from a tertiary hospital, and other projects, could contribute their observational data through adoption of this guidance. However, wholesale adoption, especially by underresourced programs, will require support, in the form of funding and training, from donors, funding agencies, national programs, and others. The demonstrable value of IPD for developing WHO MDR/RR TB treatment guidelines (*1*,*48*,*49*) and continued need for quality IPD to tackle the MDR/RR TB epidemic underscore the importance of providing this support.

The strengths of this guidance are that it draws from our extensive experience in guideline revisions, IPD collection, and IPD-MA. Furthermore, our firsthand experience receiving retrospectively collected data conforming to the data dictionary (*26*) issued during the 2018 revision of the WHO MDR/RR TB guidelines provided valuable insight into barriers to data contribution. These barriers ranged from absence of crucial clinical and patient characteristics that were never recorded (and thus could not be retrospectively obtained) to difficulty in transcribing paper records of already-collected patient data into an electronic format. This guidance should provide motivation to programs to begin prospective data collection in a standardized electronic format, which is conducive to improvements in data completeness and quality. In addition, our experiences during guideline development highlighted key areas in which data was not routinely being collected (e.g., recurrence, acquired drug resistance) and populations for whom data were scarce. This guidance should encourage the collection of such data to help answer pressing questions in these domains and populations.

The primary limitation of this guidance is that it is an initial attempt to improve practices based on experience accumulated for a very particular subtype of patients with TB. The contents of the guidance will necessarily need to evolve to the ever-changing nature of MDR/RR TB treatment and the capacities of programs to adhere to it. Successive revisions will be informed as national TB programs and other end users begin to adopt this guidance and we gain experience receiving the outputs. Finally, certain variables, such as out-of-pocket costs, lost wages, specific toxicity-related measurements (e.g., electrocardiogram, brief peripheral neuropathy screens, audiometry, liver enzymes), emergence of mental health disorders, improvement or deterioration of quality of life, and emergence of AE that are not serious or do not result in medication termination are not listed within our list of data elements. This information could be useful to patients, clinicians, and programs for specific studies, and thus could be added to local databases with care to avoid overloading data management.

Observational data will continue to play a critical role in the development of global MDR/RR TB treatment guidelines for the foreseeable future. Coordinating efforts to maximize the utility of provider experiences in MDR/RR TB is vital to improve the currently suboptimal outcomes of MDR/RR TB patients. This guidance is one key element toward achieving high-quality, comprehensive observational IPD moving forward.

Appendix Additional information quality of patient data for treatment of multidrug- or rifampin-resistant tuberculosis.

## References

[1] World Health Organization. WHO consolidated guidelines on drug-resistant tuberculosis treatment; 2019 [cited 2019 Nov 3]. https://www.who.int/tb/publications/2019/consolidated-guidelines-drug-resistant-TB-treatment 30946559

[2] Collaborative Group for the Meta-Analysis of Individual Patient Data in MDR-TB treatment–2017, Ahmad N, Ahuja SD, Akkerman OW, Alffenaar J-WC, Anderson LF, et al. Treatment correlates of successful outcomes in pulmonary multidrug-resistant tuberculosis: an individual patient data meta-analysis. Lancet. 2018;392:821–34. 10.1016/S0140-6736(18)31644-1 30215381 PMC6463280

[3] Ruswa N, Mavhunga F, Roscoe JC, Beukes A, Shipiki E, van Gorkom J, et al. Second nationwide anti-tuberculosis drug resistance survey in Namibia. Int J Tuberc Lung Dis. 2019;23:858–64. 10.5588/ijtld.18.0526 31439119 PMC6815747

[4] Centers for Disease Control and Prevention (CDC). Emergence of *Mycobacterium tuberculosis* with extensive resistance to second-line drugs—worldwide, 2000-2004. MMWR Morb Mortal Wkly Rep. 2006;55:301–5. 16557213

[5] Gotham D, Fortunak J, Pozniak A, Khoo S, Cooke G, Nytko FE III, et al. Estimated generic prices for novel treatments for drug-resistant tuberculosis. J Antimicrob Chemother. 2017;72:1243–52. 10.1093/jac/dkw522 28073970

[6] Borisov SE, D’Ambrosio L, Centis R, Tiberi S, Dheda K, Alffenaar J-W, et al. Outcomes of patients with drug-resistant-tuberculosis treated with bedaquiline-containing regimens and undergoing adjunctive surgery. J Infect. 2019;78:35–9. 10.1016/j.jinf.2018.08.003 30096332

[7] Law S, Daftary A, O’Donnell M, Padayatchi N, Calzavara L, Menzies D. Interventions to improve retention-in-care and treatment adherence among patients with drug-resistant tuberculosis: a systematic review. Eur Respir J. 2019;53:1801030. 10.1183/13993003.01030-2018 30309972

[8] Zhang Y, Wu S, Xia Y, Wang N, Zhou L, Wang J, et al. Adverse events associated with treatment of multidrug-resistant tuberculosis in China: an ambispective cohort study. Med Sci Monit. 2017;23:2348–56. 10.12659/MSM.904682 28520704 PMC5444822

[9] Honeyborne I, Lipman M, Zumla A, McHugh TD. The changing treatment landscape for MDR/XDR-TB - Can current clinical trials revolutionise and inform a brave new world? Int J Infect Dis. 2019;80S:S23–8.Error! Hyperlink reference not valid. 10.1016/j.ijid.2019.02.006 30776547

[10] von Groote-Bidlingmaier F, Patientia R, Sanchez E, Balanag V Jr, Ticona E, Segura P, et al. Efficacy and safety of delamanid in combination with an optimised background regimen for treatment of multidrug-resistant tuberculosis: a multicentre, randomised, double-blind, placebo-controlled, parallel group phase 3 trial. Lancet Respir Med. 2019;7:249–59. 10.1016/S2213-2600(18)30426-0 30630778

[11] Lee M, Lee J, Carroll MW, Choi H, Min S, Song T, et al. Linezolid for treatment of chronic extensively drug-resistant tuberculosis. N Engl J Med. 2012;367:1508–18. 10.1056/NEJMoa1201964 23075177 PMC3814175

[12] Diacon AH, Donald PR, Pym A, Grobusch M, Patientia RF, Mahanyele R, et al. Randomized pilot trial of eight weeks of bedaquiline (TMC207) treatment for multidrug-resistant tuberculosis: long-term outcome, tolerability, and effect on emergence of drug resistance. Antimicrob Agents Chemother. 2012;56:3271–6. 10.1128/AAC.06126-11 22391540 PMC3370813

[13] Tang S, Yao L, Hao X, Zhang X, Liu G, Liu X, et al. Efficacy, safety and tolerability of linezolid for the treatment of XDR-TB: a study in China. Eur Respir J. 2015;45:161–70. 10.1183/09031936.00035114 25234807

[14] Tang S, Yao L, Hao X, Liu Y, Zeng L, Liu G, et al. Clofazimine for the treatment of multidrug-resistant tuberculosis: prospective, multicenter, randomized controlled study in China. Clin Infect Dis. 2015;60:1361–7. 25605283 10.1093/cid/civ027

[15] Resist-TB. Clinical trial progress report; 2019 [cited 2019 May 15]. http://www.resisttb.org/?page_id=1602

[16] World Health Organization. Global tuberculosis report: 2018. 2019 [cited 2019 Nov 3]. https://apps.who.int/iris/bitstream/handle/10665/274453/9789241565646-eng.pdf

[17] World Health Organization. STOP TB. Contributing to health system strengthening: guiding principles for national tuberculosis programmes. 2008 [cited 2019 Nov 3]. https://www.who.int/tb/publications/tb-national-policy 24921118

[18] Franke MF, Rodriguez CA, Mitnick CD. Causal inference in tuberculosis treatment studies: bias considerations and data needs. Int J Tuberc Lung Dis. 2019;23:960–1. 10.5588/ijtld.19.0037 31533889

[19] Rodriguez CA, Mitnick CD, Franke MF. Value of observational data for multidrug-resistant tuberculosis. Lancet Infect Dis. 2019;19:930–1. 10.1016/S1473-3099(19)30424-4 31478514

[20] Guyatt GH, Oxman AD, Vist GE, Kunz R, Falck-Ytter Y, Alonso-Coello P, et al.; GRADE Working Group. GRADE: an emerging consensus on rating quality of evidence and strength of recommendations. BMJ. 2008;336:924–6. 10.1136/bmj.39489.470347.AD 18436948 PMC2335261

[21] Ahuja SD, Ashkin D, Avendano M, Banerjee R, Bauer M, Bayona JN, et al.; Collaborative Group for Meta-Analysis of Individual Patient Data in MDR-TB. Multidrug resistant pulmonary tuberculosis treatment regimens and patient outcomes: an individual patient data meta-analysis of 9,153 patients. PLoS Med. 2012;9:e1001300. 10.1371/journal.pmed.1001300 22952439 PMC3429397

[22] Falzon D, Gandhi N, Migliori GB, Sotgiu G, Cox HS, Holtz TH, et al.; Collaborative Group for Meta-Analysis of Individual Patient Data in MDR-TB. Resistance to fluoroquinolones and second-line injectable drugs: impact on multidrug-resistant TB outcomes. Eur Respir J. 2013;42:156–68. 10.1183/09031936.00134712 23100499 PMC4487776

[23] Ahmad Khan F, Salim MAH, du Cros P, Casas EC, Khamraev A, Sikhondze W, et al. Effectiveness and safety of standardised shorter regimens for multidrug-resistant tuberculosis: individual patient data and aggregate data meta-analyses. Eur Respir J. 2017;50:1700061. 10.1183/13993003.00061-2017 28751411

[24] Fregonese F, Ahuja SD, Akkerman OW, Arakaki-Sanchez D, Ayakaka I, Baghaei P, et al. Comparison of different treatments for isoniazid-resistant tuberculosis: an individual patient data meta-analysis. Lancet Respir Med. 2018;6:265–75. 10.1016/S2213-2600(18)30078-X 29595509 PMC9017096

[25] Harausz EP, Garcia-Prats AJ, Law S, Schaaf HS, Kredo T, Seddon JA, et al.; Collaborative Group for Meta-Analysis of Paediatric Individual Patient Data in MDR-TB. Treatment and outcomes in children with multidrug-resistant tuberculosis: A systematic review and individual patient data meta-analysis. PLoS Med. 2018;15:e1002591. 10.1371/journal.pmed.1002591 29995958 PMC6040687

[26] World Health Organization. Public call for individual patient data on treatment of rifampicin and multidrug-resistant (MDR/RR-TB) tuberculosis; 2018 [cited 2019 May 15]. http://www.who.int/tb/features_archive/public_call_treatment_RR_MDR_TB

[27] Getahun H, Kittikraisak W, Heilig CM, Corbett EL, Ayles H, Cain KP, et al. Development of a standardized screening rule for tuberculosis in people living with HIV in resource-constrained settings: individual participant data meta-analysis of observational studies. PLoS Med. 2011;8:e1000391. 10.1371/journal.pmed.1000391 21267059 PMC3022524

[28] Schöttker B, Jorde R, Peasey A, Thorand B, Jansen EHJM, Groot L, et al.; Consortium on Health and Ageing: Network of Cohorts in Europe and the United States. Vitamin D and mortality: meta-analysis of individual participant data from a large consortium of cohort studies from Europe and the United States. BMJ. 2014;348(jun17 16):g3656. 10.1136/bmj.g3656 24938302 PMC4061380

[29] Ben-Shlomo Y, Spears M, Boustred C, May M, Anderson SG, Benjamin EJ, et al. Aortic pulse wave velocity improves cardiovascular event prediction: an individual participant meta-analysis of prospective observational data from 17,635 subjects. J Am Coll Cardiol. 2014;63:636–46. 10.1016/j.jacc.2013.09.063 24239664 PMC4401072

[30] Morrison CS, Chen P-L, Kwok C, Baeten JM, Brown J, Crook AM, et al. Hormonal contraception and the risk of HIV acquisition: an individual participant data meta-analysis. PLoS Med. 2015;12:e1001778. 10.1371/journal.pmed.1001778 25612136 PMC4303292

[31] Yassin MA, Jaramillo E, Wandwalo E, Falzon D, Scardigli A, Kunii O, et al. Investing in a novel shorter treatment regimen for multidrug-resistant tuberculosis: to be repeated. Eur Respir J. 2017;49:1700081. 10.1183/13993003.00081-2017 28331045

[32] Migliori GB, Sotgiu G, Gandhi NR, Falzon D, DeRiemer K, Centis R, et al.; “The Collaborative Group for Meta-Analysis of Individual Patient Data in MDR-TB”. Drug resistance beyond extensively drug-resistant tuberculosis: individual patient data meta-analysis. Eur Respir J. 2013;42:169–79. 10.1183/09031936.00136312 23060633 PMC4498806

[33] Bastos ML, Hussain H, Weyer K, Garcia-Garcia L, Leimane V, Leung CC, et al.; Collaborative Group for Meta-analysis of Individual Patient Data in MDR-TB. Treatment outcomes of patients with multidrug-resistant and extensively drug-resistant tuberculosis according to drug susceptibility testing to first- and second-line drugs: an individual patient data meta-analysis. Clin Infect Dis. 2014;59:1364–74. 10.1093/cid/ciu619 25097082 PMC4296130

[34] Fox GJ, Mitnick CD, Benedetti A, Chan ED, Becerra M, Chiang C-Y, et al.; Collaborative Group for Meta-Analysis of Individual Patient Data in MDR-TB. Surgery as an adjunctive treatment for multidrug-resistant tuberculosis: an individual patient data metaanalysis. Clin Infect Dis. 2016;62:887–95. 10.1093/cid/ciw002 26757804

[35] World Health Organization. WHO TB Supranational Reference Laboratory Network: TB diagnostics and laboratory strengthening; 2014 [cited 2019 Oct 16]. https://who.int/tb/areas-of-work/laboratory/srl-network

[36] World Health Organization. Framework for conducting reviews of tuberculosis programmes. 2014 [cited 2019 Nov 3]. https://www.who.int/tb/publications/framework-tb-programme-reviews 25473711

[37] District Health Information Software. DHIS2 overview; 2019 [cited 2019 May 15]. https://www.dhis2.org/overview

[38] International Council for Harmonisation of Technical Requirements for Pharmaceuticals for Human Use. MedDRA: introductory guide version 21.0. Geneva: The Council; 2018.

[39] Laserson KF, Thorpe LE, Leimane V, Weyer K, Mitnick CD, Riekstina V, et al. Speaking the same language: treatment outcome definitions for multidrug-resistant tuberculosis. Int J Tuberc Lung Dis. 2005;9:640–5. 15971391

[40] World Health Organization. Definitions and reporting framework for tuberculosis: 2013 revision. 2014 [cited 2019 Nov 3]. https://www.who.int/tb/publications/definitions

[41] Bernaards CM, Twisk JW, Snel J, Van Mechelen W, Kemper HC. Is calculating pack-years retrospectively a valid method to estimate life-time tobacco smoking? A comparison between prospectively calculated pack-years and retrospectively calculated pack-years. Addiction. 2001;96:1653–61. 10.1046/j.1360-0443.2001.9611165311.x 11784461

[42] Kurbatova EV, Cegielski JP, Lienhardt C, Akksilp R, Bayona J, Becerra MC, et al. Sputum culture conversion as a prognostic marker for end-of-treatment outcome in patients with multidrug-resistant tuberculosis: a secondary analysis of data from two observational cohort studies. Lancet Respir Med. 2015;3:201–9. 10.1016/S2213-2600(15)00036-3 25726085 PMC4401426

[43] Halleux CM, Falzon D, Merle C, Jaramillo E, Mirzayev F, Olliaro P, et al. The World Health Organization global aDSM database: generating evidence on the safety of new treatment regimens for drug-resistant tuberculosis. Eur Respir J. 2018;51:1701643. 10.1183/13993003.01643-2017 29567719

[44] National Cancer Institute. Common Terminology Criteria for Adverse Events (CTCAE), v4.0; 2009 [cited 2019 Nov 3]. https://www.eortc.be/services/doc/ctc/CTCAE_4.03_2010-06-14_QuickReference_5x7.pdf

[45] USAID, Systems for Improved Access to Pharmaceuticals and Services. Pharmacovigilance Monitoring System (PViMS). Arlington (TX): Management Sciences for Health; 2015.

[46] International Council of Nurses, Curry International Tuberculosis Center. Nursing guide for managing side effects to drug-resistant TB treatment. Geneva: The Council; 2018.

[47] Migliori GB; Global Tuberculosis Network (GTN). Evolution of programmatic definitions used in tuberculosis prevention and care. Clin Infect Dis. 2019;68:1787–9. 10.1093/cid/ciy990 30462170

[48] World Health Organization. WHO treatment guidelines for drug-resistant tuberculosis. 2016 update. 2016 [cited 2019 Nov 3]. https://apps.who.int/iris/bitstream/handle/10665/250125/9789241549639-eng.pdf

[49] World Health Organization. Guidelines for the programmatic management of drug-resistant tuberculosis—2011 update. 2011 [cited 2019 Nov 3]. https://www.who.int/tb/challenges/mdr/programmatic_guidelines_for_mdrtb 23844450

