# Supplementary material for: Improving Quality of Patient Data for Treatment of Multidrug- or Rifampin-Resistant Tuberculosis
Source: Emerg Infect Dis. 2020 Mar;26(3):e190997. doi: 10.3201/eid2603.190997 (PMC7045826; doi:10.3201/eid2603.190997)
Supplement: Appendix — Additional information quality of patient data for treatment of multidrug- or rifampin-resistant tuberculosis. [file 19-0997-Techapp-s1.pdf]

Article DOI: https://doi.org/10.3201/eid2603.190997

Improving Quality of Patient Data for Treatment of Multidrug- or Rifampin-Resistant Tuberculosis

Appendix

Data Dictionary for MDR/RR TB IPD

The tables within this section pertain to the data elements optimally preferred for collection during the conduct of observational studies or in routinely collected programmatic data, along with their requested coding to ensure uniformity across studies. Caveats and additional information on specific elements are contained within the main text of the online report.

Facility Information					
**Field**	**Variable**	**Additional Information**	**Format**	**Category Coding**	**Category Labeling**
COUNTRY	Country	Country of the primary source	Char		
TREATING_SITE	Treating Site Name	Name of the primary source	Char		
SITE_ID	Treating Site Identifier	Site ID number	Char		

Patient Identifier and Demographics					
**Field**	**Variable**	**Additional Information**	**Format**	**Category Coding**	**Category Labeling**
PATIENT_ID	Patient Identifier	Patient ID number in country database	Char		
YEAR	Year	Year of treatment start for this episode	Num ###		
AGE	Age	Age of the patient in years	Num ###		
SEX	Sex	Patient’s biologic sex at birth	Category	F	Female
				M	Male
				U	Unknown
WEIGHT	Weight	Patient’s weight in kilograms	Num ###		
HEIGHT	Height	Patient’s height in centimeters	Num ###		
BMI	Body Mass Index	Patient’s body mass index in kilograms per meters-squared	Num ###		

Patient Baseline Characteristics					
**Field**	**Variable**	**Additional Information**	**Format**	**Category Coding**	**Category Labeling**
SMOKINGSTATUS	Smoking Status	The patient's smoking status at start of treatment	Category	Current	Current Smoker
				Ex	Ex-Smoker
				Never	Never Smoker
				U	Unknown
SMOKINGPACKPERDAY	Packs Smoked Per Day	Total number of packs per day smoked at start of treatment (if current smoker)	Num ###		
SMOKINGTOTALPACKYEAR	Total Pack Years	Total number of pack years smoked (if current- or ex-smoker)	Num ###		
ALCOHOL	Alcohol Use	Does the patient drink (defined as ≥1 drink per week in men or women)	Category	Y	Yes
				N	No
				U	Unknown
ALCOHOLABUSE	Alcohol Abuse Disorder	If the patient drinks, do they meet the definition of alcohol abuse (≥14 drinks per week in men or ≥7 drinks per week in women)	Category	Y	Yes
				N	No
				U	Unknown
DM	Diabetes Mellitus	Is the patient diagnosed with diabetes?	Category	Y	Yes
				N	No
				U	Unknown
INSULINDEPENDENT	Type 1 Diabetes Mellitus	Is the patient insulin dependent (if having diabetes)?	Category	Y	Yes
				N	No
				U	Unknown
HBA1C	Hemoglobin A1c Level	Patients HbA1c measure defined in percent (%)	Num ###		
RENALFAILURE	Presence of Renal Failure	Does the patient have renal failure?	Category	Y	Yes
				N	No
				U	Unknown
HEPB	Hepatitis B	Does the patient have hepatitis B?	Category	Y	Yes
				N	No
				U	Unknown
HEPC	Hepatitis C	Does the patient have hepatitis C?	Category	Y	Yes
				N	No
				U	Unknown
OTHERLIVER	Other Liver Condition	Does the patient have liver conditions other than hepatitis B or hepatitis C?	Category	Y	Yes
				N	No
				U	Unknown
HIV	HIV	What is the patient's HIV status?	Category	Pos	Positive
				Neg	Negative
				U	Unknown
HIV_DIAGNOSISYEAR	Year HIV Diagnosed	If the patient is HIV-positive, the year HIV was diagnosed	Num ###		
CD4	CD4 Count	If the patient is HIV-positive, what is their CD4 count at treatment start (cells/μL)?	Num ###		
VIRALLOAD	Viral Load	If the patient is HIV-positive, what is their viral load at treatment start (copies/ml)	Num ###		
ART	Use of Antiretroviral Treatment	If the patient is HIV-positive, are they on antiretroviral treatment?	Category	Y	Yes
				N	No
				U	Unknown
ART_STARTYEAR	Year Antiretroviral Treatment Started	If the patient is on antiretroviral treatment, what year did they start?	Num ###		
ART_REGIMEN	Antiretroviral Treatment Regimen	What is the antiretroviral treatment regimen?<br>List each drug, separated by a comma, using the provided abbreviations with this dictionary.	Char		

Previous Treatment Information

Field	Variable	Additional Information	Format	Category Coding	Category Labeling
PASTTX	Previous Treatment	Has the patient ever received tuberculosis treatment for >30 d?	Category	Y	Yes
				N	No
RECEIVEDFLD	Previous Treatment with First-Line Drugs	If the patient has received previous tuberculosis treatment, was treatment with first-line drugs given for >30 d?	Category	Y	Yes
				N	No
RECEIVEDSLD	Previous Treatment with Second-Line Drugs	If the patient has received previous tuberculosis treatment, was treatment with second-line drugs given for >30 d?	Category	Y	Yes
				N	No
YEARPASTTX1*	Year of Most Recent Previous Treatment	The year the patient most recently received previous tuberculosis treatment	Num ###		
REGIMENPASTTX1*	Regimen Used for Most Recent Previous Treatment	The drug-regimen given to the patient during the most recent previous tuberculosis treatment. List each drug, separated by a comma, using the provided abbreviations with this dictionary.	Char		
OUTPASTTX1*	End-of-Treatment Outcome for Most Recent Previous Treatment	The end-of-treatment outcome recorded for the patient at the end of their most recent previous tuberculosis treatment.	Category	Cure	Cure
				Complete	Completed Treatment
				Fail	Treatment Failure
				Lost	Lost to Follow-up
				U	Unknown
YEARPASTTX2*	Year of Second-Most Recent Previous Treatment	The year the patient received previous tuberculosis treatment for their second-most recent treatment episode.	Num ###		
REGIMENPASTTX2*	Regimen Used for Second-Most Recent Previous Treatment	The drug-regimen given to the patient during the second-most recent previous tuberculosis treatment. List each drug, separated by a comma, using the provided abbreviations with this dictionary.	Char		
OUTPASTTX2*	End-of-Treatment Outcome for Second-Most Recent Previous Treatment	The end-of-treatment outcome recorded for the patient at the end of their second-most recent previous tuberculosis treatment.	Category	Cure	Cure
				Complete	Completed Treatment
				Fail	Treatment Failure
				Lost	Lost to Follow-up
				U	Unknown

*Fields need to be completed only if previous treatment has been administered.

Disease Characteristics					
**Field**	**Variable**	**Additional Information**	**Format**	**Category Coding**	**Category Labeling**
DISEASE_SITE	Site of Tuberculosis Disease	The site of tuberculosis disease diagnosed in the patient	Category	PTB	
				EPTB	
				Both	
EXTRAPULM_SITE	Primary Site of Extrapulmonary Tuberculosis	If extrapulmonary tuberculosis is diagnosed, the primary site affected	Category	Miliary	Miliary TB
				Genital	Genitourinary TB
				CNS	Central Nervous System TB
				Periton	TB Peritonitis
				Pericar	TB Pericarditis
				Lymph	TB Lymphadenitis
				Pleural	Pleural TB
				GI	Gastrointestinal TB
				Bone	Bone TB
				Joint	Joint TB
				Other	Other
CAVITATION_BASE*	Lung Cavitation	Was there presence of lung cavitation on chest x-ray at treatment start?	Category	Y	Yes
				N	No
				U	Unknown
BILATERAL_BASE*	Bilateral Disease	Was there presence of bilateral disease on chest X-ray at treatment start?	Category	Y	Yes
				N	No
				U	Unknown
AFB_BASE	Acid-Fast Bacilli Smear Result	What was the patient's acid-fast bacilli smear result (taken $\leq$1 mo after treatment start)?<br>Consider all samples taken over this time frame and consider positive if any were positive (i.e., scanty or greater).	Category	Pos	Positive
				Neg	Negative
				Contam	Contaminated
				ND	Not Done
CULTURE_BASE	Sputum Culture Result	What was the patient's sputum culture result (taken $\leq$1 mo after treatment start)?<br>Consider all samples taken over this frame and consider positive if any were positive.	Category	Pos	Positive
				Neg	Negative
				Contam	Contaminated
				ND	Not Done
CULTUREMEDIA	Culture Media Used	If culture was done, what media was used for the result reported?	Category	Solid	Solid Media
				Liquid	Liquid Media
*Baseline refers to any evidence of cavitation or bilateral disease within 30 d of treatment start.					

**Genotypic DST**					
**Field***	**Variable**	**Additional Information**	**Format**	**Category Coding**	**Category Labeling**
GENOTYPIC_USED	Genotypic DST Use	Were genotypic DST techniques used?	Category	Y	Yes
				N	No
XPERT_BASE	Gene Xpert Used	Was Gene Xpert used for diagnosis?	Category	Y	Yes
				N	No
DATE_XPERT	Date of Gene Xpert	Date of Gene Xpert used for diagnosis <mm/dd/yy>	Date		
XPERT_MTBRESULT_BASE	Gene Xpert MTB Result	What was the result for MTB on Gene Xpert?	Category	Pos	Positive
				Neg	Negative
				Contam	Contaminated
XPERT_RIFRESULT_BASE	Gene Xpert Rifampin Resistance Result	What was the result for rifampin resistance on Gene Xpert?	Category	R	Resistant
				S	Susceptible
				Contam	Contaminated
FIRSTLINE_LPA_BASE	First-Line LPA Used	Was first-line LPA used after TB diagnosis?	Category	Y	Yes
				N	No
DATE_FIRSTLINE_LPA	Date of First-Line LPA	Date of first-line LPA used after TB diagnosis <mm/dd/yy>	Date		
FIRSTLINE_LPA_MTB_BASE	First-Line LPA MTB Result	What was the result for MTB on first-line LPA?	Category	Pos	Positive
				Neg	Negative
				Contam	Contaminated
FIRSTLINE_LPA_H_BASE	First-Line LPA Isoniazid Resistance Result	What was the result for isoniazid resistance on first-line LPA?	Category	R	Resistant
				S	Susceptible
				Contam	Contaminated
FIRSTLINE_LPA_R_BASE	First-Line LPA Rifampin Resistance Result	What was the result for rifampin resistance on first-line LPA?	Category	R	Resistant
				S	Susceptible
				Contam	Contaminated
SECONDLINE_LPA_BASE	Second-Line LPA Used	Was second-line LPA performed after TB diagnosis?	Category	Y	Yes
				N	No
DATE_SECONDLINE_LPA	Date of Second-Line LPA	Date of second-line LPA used after TB diagnosis <mm/dd/yy>	Date		
SECONDLINE_LPA_MTB_BASE	Second-Line LPA MTB Result	What was the result for MTB on second-line LPA?	Category	Pos	Positive
				Neg	Negative
				Contam	Contaminated
SECONDLINE_LPA_SLI_BASE	Second-Line LPA Second-Line Injectable Resistance Result	What was the result for second-line injectable resistance on second-line LPA?	Category	R	Resistant
				S	Susceptible
				Contam	Contaminated
SECONDLINE_LPA_FQ_BASE	Second-Line LPA Fluoroquinolone Resistance Result	What was the result for fluoroquinolone resistance on second-line LPA?	Category	R	Resistant
				S	Susceptible
				Contam	Contaminated

*Baseline DST refers to any sample taken within 90 d of treatment start, up to 30 d after treatment start. Every effort should be made to have reliable DST results; if genotypic tests are not used, phenotypic tests should be performed. If genotypic techniques for detection other than those listed in this table are in use (e.g., *pncA* for pyrazinamide), they may be appended to this section in a similar format (e.g., Test Done, Date of Test, Results of Test).

Phenotypic DST					
**Field***	**Variable**	**Additional Information**	**Format**	**Category Coding**	**Category Labeling**
PHENODST	Phenotypic DST Done	Was phenotypic DST performed?	Category	Y	Yes
				N	No
DATE_PHENODST	Date of Phenotypic DST	Date of phenotypic DST done after TB diagnosis <mm/dd/yy>	Date		
DST_H_BASE	Isoniazid Resistance Result	What was the result for isoniazid resistance (MIC >0.1–0.2 μg/ml on MGIT) on phenotypic DST?	Category	R	Resistant
				S	Susceptible
				Contam	Contaminated
				ND	Not Done
DST_HIGHH_BASE	High-Level Isoniazid Resistance Result	What was the result for high-level isoniazid resistance (MIC >1–2 μg/ml on MGIT) on phenotypic DST?	Category	R	Resistant
				S	Susceptible
				Contam	Contaminated
				ND	Not Done
DST_R_BASE	Rifampin Resistance Result	What was the result for rifampin resistance on phenotypic DST?	Category	R	Resistant
				S	Susceptible
				Contam	Contaminated
				ND	Not Done
DST_E_BASE	Ethambutol Resistance Result	What was the result for ethambutol resistance on phenotypic DST?	Category	R	Resistant
				S	Susceptible
				Contam	Contaminated
				ND	Not Done
DST_Z_BASE	Pyrazinamide Resistance Result	What was the result for pyrazinamide resistance on phenotypic DST?	Category	R	Resistant
				S	Susceptible
				Contam	Contaminated
				ND	Not Done
DST_AM_BASE	Amikacin Resistance Result	What was the result for amikacin resistance on phenotypic DST?	Category	R	Resistant
				S	Susceptible
				Contam	Contaminated
				ND	Not Done
DST_KM_BASE	Kanamycin Resistance Result	What was the result for kanamycin resistance on phenotypic DST?	Category	R	Resistant
				S	Susceptible
				Contam	Contaminated
				ND	Not Done
DST_CM_BASE	Capreomycin Resistance Result	What was the result for capreomycin resistance on phenotypic DST?	Category	R	Resistant
				S	Susceptible
				Contam	Contaminated
				ND	Not Done
DST_OFX_BASE	Ofloxacin Resistance Result	What was the result for ofloxacin resistance on phenotypic DST?	Category	R	Resistant
				S	Susceptible
				Contam	Contaminated
				ND	Not Done
DST_CFX_BASE	Ciprofloxacin Resistance Result	What was the result for ciprofloxacin resistance on phenotypic DST?	Category	R	Resistant
				S	Susceptible
				Contam	Contaminated
				ND	Not Done
DST_MFX_BASE	Moxifloxacin Resistance Result	What was the result for moxifloxacin resistance on phenotypic DST?	Category	R	Resistant
				S	Susceptible
				Contam	Contaminated
				ND	Not Done
DST_LFX_BASE	Levofloxacin Resistance Result	What was the result for levofloxacin resistance on phenotypic DST?	Category	R	Resistant
				S	Susceptible
				Contam	Contaminated
				ND	Not Done
DST_S_BASE	Streptomycin Resistance Result	What was the result for streptomycin resistance on phenotypic DST?	Category	R	Resistant
				S	Susceptible
				Contam	Contaminated
				ND	Not Done
DST_ETO_BASE	Ethionamide Resistance Result	What was the result for ethionamide resistance on phenotypic DST?	Category	R	Resistant
				S	Susceptible
				Contam	Contaminated
				ND	Not Done
DST_PTO_BASE	Prothionamide Resistance Result	What was the result for prothionamide resistance on phenotypic DST?	Category	R	Resistant
				S	Susceptible
				Contam	Contaminated

Phenotypic DST					
**Field***	**Variable**	**Additional Information**	**Format**	**Category Coding**	**Category Labeling**
				ND	Not Done
DST_CS_BASE	Cycloserine Resistance Result	What was the result for cycloserine resistance on phenotypic DST?	Category	R	Resistant
				S	Susceptible
				Contam	Contaminated
				ND	Not Done
DST_TRD_BASE	Terizidone Resistance Result	What was the result for terizidone resistance on phenotypic DST?	Category	R	Resistant
				S	Susceptible
				Contam	Contaminated
				ND	Not Done
DST_PAS_BASE	Para-Amino-Salicylic Acid Resistance Result	What was the result for para-amino-salicylic acid resistance on phenotypic DST?	Category	R	Resistant
				S	Susceptible
				Contam	Contaminated
				ND	Not Done
DST_LZD_BASE	Linezolid Resistance Result	What was the result for linezolid resistance on phenotypic DST?	Category	R	Resistant
				S	Susceptible
				Contam	Contaminated
				ND	Not Done
DST_CFZ_BASE	Clofazimine Resistance Result	What was the result for clofazimine resistance on phenotypic DST?	Category	R	Resistant
				S	Susceptible
				Contam	Contaminated
				ND	Not Done
DST_BDQ_BASE	Bedaquiline Resistance Result	What was the result for bedaquiline resistance on phenotypic DST?	Category	R	Resistant
				S	Susceptible
				Contam	Contaminated
				ND	Not Done
DST_DLM_BASE	Delamanid Resistance Result	What was the result for delamanid resistance on phenotypic DST?	Category	R	Resistant
				S	Susceptible
				Contam	Contaminated
				ND	Not Done
*Baseline DST refers to any sample taken within 90 d of treatment start, up to 30 d after treatment start. Additional drugs for which phenotypic DST is available can be reported (e.g., Pretomanid). Within all shared data, the method and critical concentration used for each drug must be recorded.					

Follow-Up DST and Acquired Drug Resistance					
**Field**	**Variable**	**Additional Information**	**Format**	**Category Coding**	**Category Labeling**
FOLLOWUP_DST	Follow-up DST Performed	Was there follow-up DST performed?	Category	Y	Yes
				N	No
FOLLOWUPDST1_DATE*	Date of First Follow-up DST	Date of first follow-up DST <mm/dd/yy>	Date		
FOLLOWUPDST_RES1	Resistant Isolates on First Follow-up DST	List newly discovered resistances not found on baseline DST, due to missingness or baseline susceptibility. If none discovered, list “no change in DST.” List each drug, separated by a comma, using the provided abbreviations with this dictionary.	Char		
FOLLOWUPDST_SUS1	Susceptible Isolates on First Follow-up DST	List newly discovered susceptible drugs not found on baseline DST, due to missingness or baseline resistance. If none discovered, list “no change in DST.” List each drug, separated by a comma, using the provided abbreviations with this dictionary.	Char		
ACQUIRED_RESISTANCE†	Acquired Drug Resistance	List the drugs that the strain was shown to acquire resistance to during any follow-up DST (defined as previously identified susceptibility and subsequent resistance on follow-up DST). List each drug, separated by a comma, using the provided abbreviations with this dictionary.	Char		

*Additional follow-up DST results can be entered following a similar format.
†Acquired resistance can be reported in a separate row but is not necessary as it can be calculated by the data analyst with the above collected data.

Regimen Information*					
**Field**	**Variable**	**Additional Information**	**Format**	**Category Coding**	**Category Labeling**
STARTINGREGIMENTYPE	Regimen Type at Start of Treatment	List the starting regimen type: short (intended duration ≤12 mo) or long (intended duration ≥18 mo)	Category	Short	Short Regimen
				Long	Long Regimen
TXSTART_DATE	Treatment Start Date	Date of second-line drug initiation in this treatment episode <mm/dd/yy>	Date		
INITIAL_REGIMEN	Starting Treatment Regimen	List the drugs the patient is on at the start of treatment. List each drug, separated by a comma, using the provided abbreviations with this dictionary.	Char		
H_START	Isoniazid Start Date	Date standard-dose isoniazid was introduced into the patient's regimen. <mm/dd/yy>	Date		
H_STOP	Isoniazid End Date	Date standard-dose isoniazid was permanently removed from the patient's regimen <mm/dd/yy>	Date		
HIGHH_START	High-Dose Isoniazid Start Date	Date high-dose isoniazid was introduced into the patient's regimen. <mm/dd/yy>	Date		
HIGHH_STOP	High-Dose Isoniazid End Date	Date high-dose isoniazid was permanently removed from the patient's regimen <mm/dd/yy>	Date		
E_START	Ethambutol Start Date	Date ethambutol was introduced into the patient's regimen. <mm/dd/yy>	Date		
E_STOP	Ethambutol End Date	Date ethambutol was permanently removed from the patient's regimen <mm/dd/yy>	Date		
Z_START	Pyrazinamide Start Date	Date pyrazinamide was introduced into the patient's regimen. <mm/dd/yy>	Date		
Z_STOP	Pyrazinamide End Date	Date pyrazinamide was permanently removed from the patient's regimen <mm/dd/yy>	Date		
S_START	Streptomycin Start Date	Date streptomycin isoniazid was introduced into the patient's regimen. <mm/dd/yy>	Date		
S_STOP	Streptomycin End Date	Date streptomycin was permanently removed from the patient's regimen <mm/dd/yy>	Date		
RFB_START	Rifabutin Start Date	Date rifabutin was introduced into the patient's regimen. <mm/dd/yy>	Date		
RFB_STOP	Rifabutin End Date	Date rifabutin was permanently removed from the patient's regimen <mm/dd/yy>	Date		
S_START	Streptomycin Start Date	Date streptomycin was introduced into the patient's regimen. <mm/dd/yy>	Date		
S_STOP	Streptomycin End Date	Date streptomycin was permanently removed from the patient's regimen <mm/dd/yy>	Date		
AM_START	Amikacin Start Date	Date amikacin was introduced into the patient's regimen. <mm/dd/yy>	Date		
AM_STOP	Amikacin End Date	Date amikacin was permanently removed from the patient's regimen	Date		

Regimen Information*					
**Field**	**Variable**	**Additional Information**	**Format**	**Category Coding**	**Category Labeling**
		<mm/dd/yy>			
KM_START	Kanamycin Start Date	Date kanamycin was introduced into the patient's regimen. <mm/dd/yy>	Date		
KM_STOP	Kanamycin End Date	Date kanamycin was permanently removed from the patient's regimen <mm/dd/yy>	Date		
CM_START	Capreomycin Start Date	Date capreomycin was introduced into the patient's regimen. <mm/dd/yy>	Date		
CM_STOP	Capreomycin End Date	Date capreomycin was permanently removed from the patient's regimen <mm/dd/yy>	Date		
OFX_START	Ofloxacin Start Date	Date ofloxacin was introduced into the patient's regimen. <mm/dd/yy>	Date		
OFX_STOP	Ofloxacin End Date	Date ofloxacin was permanently removed from the patient's regimen <mm/dd/yy>	Date		
CFX_START	Ciprofloxacin Start Date	Date ciprofloxacin was introduced into the patient's regimen. <mm/dd/yy>	Date		
CFX_STOP	Ciprofloxacin End Date	Date ciprofloxacin was permanently removed from the patient's regimen <mm/dd/yy>	Date		
MFX_START	Moxifloxacin Start Date	Date moxifloxacin was introduced into the patient's regimen. <mm/dd/yy>	Date		
MFX_STOP	Moxifloxacin End Date	Date moxifloxacin was permanently removed from the patient's regimen <mm/dd/yy>	Date		
LFX_START	Levofloxacin Start Date	Date levofloxacin was introduced into the patient's regimen. <mm/dd/yy>	Date		
LFX_STOP	Levofloxacin End Date	Date levofloxacin was permanently removed from the patient's regimen <mm/dd/yy>	Date		
GFX_START	Gatifloxacin Start Date	Date gatifloxacin was introduced into the patient's regimen. <mm/dd/yy>	Date		
GFX_STOP	Gatifloxacin End Date	Date gatifloxacin was permanently removed from the patient's regimen <mm/dd/yy>	Date		
SFX_START	Sparfloxacin Start Date	Date sparfloxacin was introduced into the patient's regimen. <mm/dd/yy>	Date		
SFX_STOP	Sparfloxacin End Date	Date sparfloxacin was permanently removed from the patient's regimen <mm/dd/yy>	Date		
ETO_START	Ethionamide Start Date	Date ethionamide was introduced into the patient's regimen. <mm/dd/yy>	Date		
ETO_STOP	Ethionamide End Date	Date ethionamide was permanently removed from the patient's regimen <mm/dd/yy>	Date		
PTO_START	Prothionamide Start Date	Date prothionamide was introduced into the patient's regimen. <mm/dd/yy>	Date		
PTO_STOP	Prothionamide End Date	Date prothionamide was permanently removed from the patient's regimen <mm/dd/yy>	Date		
CS_START	Cycloserine Start Date	Date cycloserine was introduced into the patient's regimen. <mm/dd/yy>	Date		

Regimen Information*					
**Field**	**Variable**	**Additional Information**	**Format**	**Category Coding**	**Category Labeling**
CS_STOP	Cycloserine End Date	Date cycloserine was permanently removed from the patient’s regimen <mm/dd/yy>	Date		
TRD_START	Terizidone Start Date	Date terizidone was introduced into the patient’s regimen. <mm/dd/yy>	Date		
TRD_STOP	Terizidone End Date	Date terizidone was permanently removed from the patient’s regimen <mm/dd/yy>	Date		
PAS_START	Para-Aminosalicylic Acid Start Date	Date para-aminosalicylic acid was introduced into the patient’s regimen. <mm/dd/yy>	Date		
PAS_STOP	Para-Aminosalicylic Acid End Date	Date para-aminosalicylic acid was permanently removed from the patient’s regimen <mm/dd/yy>	Date		
LZD_START	Linezolid Start Date	Date linezolid was introduced into the patient’s regimen. <mm/dd/yy>	Date		
LZD_STOP	Linezolid End Date	Date linezolid was permanently removed from the patient’s regimen <mm/dd/yy>	Date		
CFZ_START	Clofazimine Start Date	Date clofazimine was introduced into the patient’s regimen. <mm/dd/yy>	Date		
CFZ_STOP	Clofazimine End Date	Date clofazimine was permanently removed from the patient’s regimen <mm/dd/yy>	Date		
AMXCLV_START	Amoxicillin and Clavulanic Acid Start Date	Date amoxicillin and clavulanic acid was introduced into the patient’s regimen. <mm/dd/yy>	Date		
AMXCLV_STOP	Amoxicillin and Clavulanic Acid End Date	Date amoxicillin and clavulanic acid was permanently removed from the patient’s regimen <mm/dd/yy>	Date		
IPM_START	Imipenem-Cilastatin Start Date	Date imipenem-cilastatin was introduced into the patient’s regimen. <mm/dd/yy>	Date		
IPM_STOP	Imipenem-Cilastatin End Date	Date imipenem-cilastatin was permanently removed from the patient’s regimen <mm/dd/yy>	Date		
MPM_START	Meropenem Start Date	Date meropenem was introduced into the patient’s regimen. <mm/dd/yy>	Date		
MPM_STOP	Meropenem End Date	Date meropenem was permanently removed from the patient’s regimen <mm/dd/yy>	Date		
BDQ_START	Bedaquiline Start Date	Date bedaquiline was introduced into the patient’s regimen. <mm/dd/yy>	Date		
BDQ_STOP	Bedaquiline End Date	Date bedaquiline was permanently removed from the patient’s regimen <mm/dd/yy>	Date		
DLM_START	Delamanid Start Date	Date delamanid was introduced into the patient’s regimen. <mm/dd/yy>	Date		
DLM_STOP	Delamanid End Date	Date delamanid was permanently removed from the patient’s regimen <mm/dd/yy>	Date		
PA_START	Pretomanid Start Date	Date pretomanid was introduced into the patient’s regimen. <mm/dd/yy>	Date		

Regimen Information*					
**Field**	**Variable**	**Additional Information**	**Format**	**Category Coding**	**Category Labeling**
PA_STOP	Pretomanid End Date	Date pretomanid was permanently removed from the patient’s regimen <mm/dd/yy>	Date		
PCZ_START	Perchlozone Start Date	Date perchlozone was introduced into the patient’s regimen. <mm/dd/yy>	Date		
PCZ_STOP	Perchlozone End Date	Date perchlozone was permanently removed from the patient’s regimen <mm/dd/yy>	Date		
TXEND_DATE	Treatment End Date	Date treatment ended in this treatment episode <mm/dd/yy>	Date		
DURATION_CHANGE	Intended Duration of Regimen Changed	If the patient started on a short regimen, did they switch to a long regimen?	Category	Y	Yes
				N	No
CHANGE_DATE	Date of Regimen Duration Change	The date the patient changed from a short regimen to a long regimen <mm/dd/yy>	Date		
CHANGE_REASON	Reason the Regimen Duration Changed	What was the reason the regimen duration changed? This may include: in response to drug susceptibility testing, treatment non-response, drug availability, drug tolerability, or other.	Category	DST	Drug Resistance
				NoResp	Non-Response
				AE	Tolerability
				Avail	Drug Availability
				Other	Other
*For drugs not used in the regimen, their coding can remain blank. Stop dates must refer to the date that the drug was permanently withdrawn from the regimen. New rows can be added to accommodate drugs not contained in this table.					

Treatment Information					
**Field**	**Variable**	**Additional Information**	**Format**	**Category Coding**	**Category Labeling**
TXDUR_MONTHS	Treatment Duration	Total number of days of treatment, from first to last dose taken	Num ###		
DOT	Directly Observed Therapy	Was directly observed therapy used?	Category	Y	Yes
				N	No
DOT_TYPE	Type of Directly Observed Therapy	State the type of directly observed therapy used. Virtual includes methods such as video, mobile text, or medication monitoring, among others.	Category	Comm	Community
				Hosp	Hospital
				Pharm	Pharmacy
				Virtual	Virtual
DOT_FREQUENCY	Frequency of DOT Visits	How many days per week is DOT provided to the patient (range 0–7 d)	Num ###		
SUPPORT	Patient Support Provided	What form of patient support was provided to patients? This may include support from employers (job security), nutritional support, financial support, or others. If more than one form, please select multiple.	Category	Employ	Employment
				Nutri	Nutritional
				Finance	Financial
				Other	Other
				Multi	Multiple
				None	None

Surgery and Hospitalization Information					
**Field**	**Variable**	**Additional Information**	**Format**	**Category Coding**	**Category Labeling**
SURGERY	Lung Resection Surgery	Did the patient have lung resection surgery related to MDR/RR-TB?	Category	Y	Yes
				N	No
				U	Unknown
SURGTYPE	Type of Lung Resection Surgery	What was the type of lung resection surgery?		Lobe	Lobectomy
				Pneu	Pneumonectomy
				Wedge	Wedge Resection
				Other	Other
				U	Unknown
SURG_DATE	Date of Surgery	What was the date of surgery?	Date		
HOSP	Hospitalization	Was the patient hospitalized at any point during treatment?	Category	Y	Yes
				N	No
				U	Unknown
HOSPEPISODES	Number of Hospitalization Episodes	What is the total number of hospitalization episodes during treatment?	Num ###		
HOSPDUR_DAYS	Total Hospitalization Duration	What is the total duration of hospitalization during treatment?	Num ###		

Adverse Event Information					
**Field***	**Variable**	**Additional Information**	**Format**	**Category Coding**	**Category Labeling**
AE1	First Adverse Event	Did the patient experience a serious adverse event or permanently stop the drug?	Category	SAE	Serious Adverse Event
				Perm	Permanent Stop
				Both	Both
AE1_DATE	Date of First Adverse event	What was the date of the permanent discontinuation of the drug(s)?	Date		
AE1_DRUG	Drug Responsible for First Adverse Event	List each drug, separated by a comma, using the provided abbreviations with this dictionary.	Char		
AE1_GRADE	Grade of First Adverse Event	What was the grade of the first adverse event?	Num ###		
AE1_SYSTEMORGAN	System / Organ Class Affected by First Adverse Event	Which system / organ classes were affected by the first adverse event? List each system / organ class, separated by a comma, using the list provided with this dictionary.	Char		
AE1_OUTCOME	Outcome of First Adverse Event	What was the outcome of the first adverse event?	Category	Recov	Recovered
				NoRecov	Not Recovered
				Died	Died
				U	Unknown
AE2	Second Adverse Event	Did the patient experience a serious adverse event or permanently stop the drug?	Category	SAE	Serious Adverse Event
				Perm	Permanent Stop
				Both	Both
AE2_DATE	Date of Second Adverse event	What was the date of the permanent discontinuation of the drug(s)?	Date		
AE2_DRUG	Drug Responsible for Second Adverse Event	List each drug, separated by a comma, using the provided abbreviations with this dictionary.	Char		
AE2_GRADE	Grade of Second Adverse Event	What was the grade of the second adverse event?	Num ###		
AE2_SYSTEMORGAN	System / Organ Class Affected by Second Adverse Event	Which system / organ classes were affected by the second adverse event? List each system / organ class, separated by a comma, using the list provided with this dictionary.	Char		
AE2_OUTCOME	Outcome of Second Adverse Event	What was the outcome of the second adverse event?	Category	Recov	Recovered
				NoRecov	Not Recovered
				Died	Died
				U	Unknown
AE3	Third Adverse Event	Did the patient experience a serious adverse event or permanently stop the drug?	Category	SAE	Serious Adverse Event
				Perm	Permanent Stop
				Both	Both
AE3_DATE	Date of Third Adverse event	What was the date of the permanent discontinuation of the drug(s)?	Date		
AE3_DRUG	Drug Responsible for Third Adverse Event	List each drug, separated by a comma, using the provided abbreviations with this dictionary.	Char		
AE3_GRADE	Grade of Third Adverse Event	What was the grade of the third adverse event?	Num ###		
AE3_SYSTEMORGAN	System / Organ Class Affected by Third Adverse Event	Which system / organ classes were affected by the third adverse event? List each system / organ class, separated by a comma, using the list provided with this dictionary.	Char		
AE3_OUTCOME	Outcome of Third Adverse Event	What was the outcome of the third adverse event?	Category	Recov	Recovered
				NoRecov	Not Recovered
				Died	Died
				U	Unknown
*Additional adverse event entries can be entered following the same format.					

Follow-Up Culture Results*					
**Field**	**Variable**	**Additional Information**	**Format**	**Category Coding**	**Category Labeling**
CULTURE_MONTH1	Culture Result Month 1	What is the culture result for the sputum sample tested during month 1?	Category	Pos	Positive
				Neg	Negative
				Contam	Contaminated
				ND	Not Done
CULTURE_MONTH2	Culture Result Month 2	What is the culture result for the sputum sample tested during month 2?	Category	Pos	Positive
				Neg	Negative
				Contam	Contaminated
				ND	Not Done
CULTURE_MONTH3	Culture Result Month 3	What is the culture result for the sputum sample tested during month 3?	Category	Pos	Positive
				Neg	Negative
				Contam	Contaminated
				ND	Not Done
CULTURE_MONTH4	Culture Result Month 4	What is the culture result for the sputum sample tested during month 4?	Category	Pos	Positive
				Neg	Negative
				Contam	Contaminated
				ND	Not Done
CULTURE_MONTH5	Culture Result Month 5	What is the culture result for the sputum sample tested during month 5?	Category	Pos	Positive
				Neg	Negative
				Contam	Contaminated
				ND	Not Done
CULTURE_MONTH6	Culture Result Month 6	What is the culture result for the sputum sample tested during month 6?	Category	Pos	Positive
				Neg	Negative
				Contam	Contaminated
				ND	Not Done
CULTURE_MONTH7	Culture Result Month 7	What is the culture result for the sputum sample tested during month 7?	Category	Pos	Positive
				Neg	Negative
				Contam	Contaminated
				ND	Not Done
CULTURE_MONTH8	Culture Result Month 8	What is the culture result for the sputum sample tested during month 8?	Category	Pos	Positive
				Neg	Negative
				Contam	Contaminated
				ND	Not Done
CULTURE_MONTH9	Culture Result Month 9	What is the culture result for the sputum sample tested during month 9?	Category	Pos	Positive
				Neg	Negative
				Contam	Contaminated
				ND	Not Done
CULTURE_MONTH10	Culture Result Month 10	What is the culture result for the sputum sample tested during month 10?	Category	Pos	Positive
				Neg	Negative
				Contam	Contaminated
				ND	Not Done
CULTURE_MONTH11	Culture Result Month 11	What is the culture result for the sputum sample tested during month 11?	Category	Pos	Positive
				Neg	Negative
				Contam	Contaminated
				ND	Not Done
CULTURE_MONTH12	Culture Result Month 12	What is the culture result for the sputum sample tested during month 12?	Category	Pos	Positive
				Neg	Negative
				Contam	Contaminated
				ND	Not Done
CULTURE_MONTH13	Culture Result Month 13	What is the culture result for the sputum sample tested during month 13?	Category	Pos	Positive
				Neg	Negative
				Contam	Contaminated
				ND	Not Done
CULTURE_MONTH14	Culture Result Month 14	What is the culture result for the sputum sample tested during month 14?	Category	Pos	Positive
				Neg	Negative
				Contam	Contaminated
				ND	Not Done
CULTURE_MONTH15	Culture Result Month 15	What is the culture result for the sputum sample tested during month 15?	Category	Pos	Positive
				Neg	Negative
				Contam	Contaminated
				ND	Not Done
CULTURE_MONTH16	Culture Result Month 16	What is the culture result for the sputum sample tested during month 16?	Category	Pos	Positive
				Neg	Negative
				Contam	Contaminated

Follow-Up Culture Results*					
**Field**	**Variable**	**Additional Information**	**Format**	**Category Coding**	**Category Labeling**
				ND	Not Done
CULTURE_MONTH17	Culture Result Month 17	What is the culture result for the sputum sample tested during month 17?	Category	Pos	Positive
				Neg	Negative
				Contam	Contaminated
				ND	Not Done
CULTURE_MONTH18	Culture Result Month 18	What is the culture result for the sputum sample tested during month 18?	Category	Pos	Positive
				Neg	Negative
				Contam	Contaminated
				ND	Not Done
CULTURE_MONTH19	Culture Result Month 19	What is the culture result for the sputum sample tested during month 19?	Category	Pos	Positive
				Neg	Negative
				Contam	Contaminated
				ND	Not Done
CULTURE_MONTH20	Culture Result Month 20	What is the culture result for the sputum sample tested during month 20?	Category	Pos	Positive
				Neg	Negative
				Contam	Contaminated
				ND	Not Done
CULTURE_MONTH21	Culture Result Month 21	What is the culture result for the sputum sample tested during month 21?	Category	Pos	Positive
				Neg	Negative
				Contam	Contaminated
				ND	Not Done
CULTURE_MONTH22	Culture Result Month 22	What is the culture result for the sputum sample tested during month 22?	Category	Pos	Positive
				Neg	Negative
				Contam	Contaminated
				ND	Not Done
CULTURE_MONTH23	Culture Result Month 23	What is the culture result for the sputum sample tested during month 23?	Category	Pos	Positive
				Neg	Negative
				Contam	Contaminated
				ND	Not Done
CULTURE_MONTH24	Culture Result Month 24	What is the culture result for the sputum sample tested during month 24?	Category	Pos	Positive
				Neg	Negative
				Contam	Contaminated
				ND	Not Done
*Month 1 refers to the result of the sample taken between day 31 and 60 that is closest to day 31 and valid (i.e., Positive or Negative); Month 2 refers to the sample taken between day 61 and 90 that is closest to day 61 and valid (i.e., Positive or Negative); the remaining months follow the same pattern. Any MTB colonies seen should be considered positive. If multiple samples are taken on a given day, a positive-dominant approach should be taken, whereby a patient is positive if a single positive sample is found. A patient sample should only be classified as contaminated if all samples from that month were contaminated.					

Follow-Up Smear Microscopy Results*					
**Field**	**Variable**	**Additional Information**	**Format**	**Category Coding**	**Category Labeling**
SMEAR_MONTH1	Smear Result Month 1	What is the smear result for the sputum sample tested during month 1?	Category	Pos	Positive
				Neg	Negative
				Contam	Contaminated
				ND	Not Done
SMEAR_MONTH2	Smear Result Month 2	What is the smear result for the sputum sample tested during month 2?	Category	Pos	Positive
				Neg	Negative
				Contam	Contaminated
				ND	Not Done
SMEAR_MONTH3	Smear Result Month 3	What is the smear result for the sputum sample tested during month 3?	Category	Pos	Positive
				Neg	Negative
				Contam	Contaminated
				ND	Not Done
SMEAR_MONTH4	Smear Result Month 4	What is the smear result for the sputum sample tested during month 4?	Category	Pos	Positive
				Neg	Negative
				Contam	Contaminated
				ND	Not Done
SMEAR_MONTH5	Smear Result Month 5	What is the smear result for the sputum sample tested during month 5?	Category	Pos	Positive
				Neg	Negative
				Contam	Contaminated
				ND	Not Done
SMEAR_MONTH6	Smear Result Month 6	What is the smear result for the sputum sample tested during month 6?	Category	Pos	Positive
				Neg	Negative
				Contam	Contaminated
				ND	Not Done
SMEAR_MONTH7	Smear Result Month 7	What is the smear result for the sputum sample tested during month 7?	Category	Pos	Positive
				Neg	Negative
				Contam	Contaminated
				ND	Not Done
SMEAR_MONTH8	Smear Result Month 8	What is the smear result for the sputum sample tested during month 8?	Category	Pos	Positive
				Neg	Negative
				Contam	Contaminated
				ND	Not Done
SMEAR_MONTH9	Smear Result Month 9	What is the smear result for the sputum sample tested during month 9?	Category	Pos	Positive
				Neg	Negative
				Contam	Contaminated
				ND	Not Done
SMEAR_MONTH10	Smear Result Month 10	What is the smear result for the sputum sample tested during month 10?	Category	Pos	Positive
				Neg	Negative
				Contam	Contaminated
				ND	Not Done
SMEAR_MONTH11	Smear Result Month 11	What is the smear result for the sputum sample tested during month 11?	Category	Pos	Positive
				Neg	Negative
				Contam	Contaminated
				ND	Not Done
SMEAR_MONTH12	Smear Result Month 12	What is the smear result for the sputum sample tested during month 12?	Category	Pos	Positive
				Neg	Negative
				Contam	Contaminated
				ND	Not Done
SMEAR_MONTH13	Smear Result Month 13	What is the smear result for the sputum sample tested during month 13?	Category	Pos	Positive
				Neg	Negative
				Contam	Contaminated
				ND	Not Done
SMEAR_MONTH14	Smear Result Month 14	What is the smear result for the sputum sample tested during month 14?	Category	Pos	Positive
				Neg	Negative
				Contam	Contaminated
				ND	Not Done
SMEAR_MONTH15	Smear Result Month 15	What is the smear result for the sputum sample tested during month 15?	Category	Pos	Positive
				Neg	Negative
				Contam	Contaminated
				ND	Not Done
SMEAR_MONTH16	Smear Result Month 16		Category	Pos	Positive
				Neg	Negative

Follow-Up Smear Microscopy Results*					
**Field**	**Variable**	**Additional Information**	**Format**	**Category Coding**	**Category Labeling**
		What is the smear result for the sputum sample tested during month 16?		Contam	Contaminated
				ND	Not Done
SMEAR_MONTH17	Smear Result Month 17	What is the smear result for the sputum sample tested during month 17?	Category	Pos	Positive
				Neg	Negative
				Contam	Contaminated
				ND	Not Done
SMEAR_MONTH18	Smear Result Month 18	What is the smear result for the sputum sample tested during month 18?	Category	Pos	Positive
				Neg	Negative
				Contam	Contaminated
				ND	Not Done
SMEAR_MONTH19	Smear Result Month 19	What is the smear result for the sputum sample tested during month 19?	Category	Pos	Positive
				Neg	Negative
				Contam	Contaminated
				ND	Not Done
SMEAR_MONTH20	Smear Result Month 20	What is the smear result for the sputum sample tested during month 20?	Category	Pos	Positive
				Neg	Negative
				Contam	Contaminated
				ND	Not Done
SMEAR_MONTH21	Smear Result Month 21	What is the smear result for the sputum sample tested during month 21?	Category	Pos	Positive
				Neg	Negative
				Contam	Contaminated
				ND	Not Done
SMEAR_MONTH22	Smear Result Month 22	What is the smear result for the sputum sample tested during month 22?	Category	Pos	Positive
				Neg	Negative
				Contam	Contaminated
				ND	Not Done
SMEAR_MONTH23	Smear Result Month 23	What is the smear result for the sputum sample tested during month 23?	Category	Pos	Positive
				Neg	Negative
				Contam	Contaminated
				ND	Not Done
SMEAR_MONTH24	Smear Result Month 24	What is the smear result for the sputum sample tested during month 24?	Category	Pos	Positive
				Neg	Negative
				Contam	Contaminated
				ND	Not Done
*Any acid-fast bacilli seen should be considered positive. Month 1 refers to the result of the sample taken between day 31 and 60 that is closest to day 31 and valid (i.e., Positive or Negative); Month 2 refers to the sample taken between day 61 and 90 that is closest to day 61 and valid (i.e., Positive or Negative); the remaining months follow the same pattern. If multiple samples are taken within a given day, a positive-dominant approach should be taken, whereby a patient is positive if a single positive sample is found. A patient sample should be classified as contaminated only if all samples from that month were contaminated.					

Treatment Outcome Information					
**Field**	**Variable**	**Additional Information**	**Format**	**Category Coding**	**Category Labeling**
OUTCOME_DEFINITION	End-of-Treatment Outcome Definition	Specify the guideline year the outcome definition follows—this is preferably the 2013 guidelines but can follow 2005 guidelines if not available.	Category	WHO2013	2013 Definitions
				WHO2005	2005 Definitions
OUTCOME	End-of-Treatment Outcome	End of treatment outcome assigned to the patient, following the outcome year specified above.	Category	Cure	Cure
				Complete	Treatment Complete
				Fail	Treatment Failure
				Death	Death
				LTFU	Loss to Follow-Up
CULTURECONV*	Culture Conversion	Did the patient culture convert (defined as two consecutive negative cultures taken at least 28 d apart)? If the patient was culture negative at baseline, list as BaseNeg.	Category	Y	Yes
				N	No
				BaseNeg	Baseline Negative
CULTURECONV_DATE	Date of Culture Conversion	If the patient culture converted, what was the date of conversion (defined as the date of the first of the two consecutive negative cultures)?	Date		
TWOCONV	Culture Conversion by Month Two	If exact date of conversion is unknown, did culture conversion occur before the end of month two?	Category	Y	Yes
				N	No
				U	Unknown
SIXCONV	Culture Conversion by Month Six	If exact date of conversion is unknown, did culture conversion occur before the end of month six?	Category	Y	Yes
				N	No
				U	Unknown
CULTUREREV*	Culture Reversion	If patient converted or was culture negative at baseline, was there culture reversion (defined as two consecutive positive cultures taken at least 28 d apart)?	Category	Y	Yes
				N	No
				U	Unknown
CULTUREREV_DATE	Date of Culture Reversion	If patient had culture reversion, what was the date of reversion (defined as the date of the first of the two consecutive positive cultures)?	Date		
RECURRENCE_MONITORING	Post-Treatment Recurrence Monitoring	Was post-treatment monitoring for recurrence performed?	Category	Y	Yes
				N	No
RECURRENCE_FOLLOWUP_DUR	Duration of Recurrence Monitoring	What was the duration of recurrence monitoring, in months?	Num ###		
RECURRENCE_OUTCOME	Occurrence of Recurrence	Did the patient experience recurrence?	Category	Y	Yes
				N	No
RECURRENCE_DATE	Date of Recurrence	What was the date of the recurrence episode?	Date		
RELAPSE_REINFECT	Relapse or Reinfection	If resources permitted, was the recurrence classified as a true relapse or as a reinfection?	Category	Relapse	Relapse
				Reinfect	Reinfection
				U	Unknown
*These can be reported by the individual providing data or calculated by an analyst. In the instance of multiple cultures taken at the same time, a positive dominant approach should be taken, i.e., the result should be considered positive if any of the samples are positive. In the case of contaminated results, these should be discarded when calculating time to culture conversion or reversion.					

Drug Abbreviations, System/Organ Classes, and End-of-Treatment Outcome Definitions

The tables contained within this section are intended to promote standardization in coding of drugs, outcomes, and adverse events.

Tuberculosis Drug Name / Drug Class	Abbreviation
Isoniazid	H
Rifampin	R
Ethambutol	E
Pyrazinamide	Z
High Dose Isoniazid	HighH
Streptomycin	S
Rifabutin	Rfb
Amikacin	Am
Capreomycin	Cm
Kanamycin	Km
Ofloxacin	Ofx
Ciprofloxacin	Cfx
Moxifloxacin	Mfx
Levofloxacin	Lfx
Gatifloxacin	Gfx
Sparfloxacin	Sfx
Ethionamide	Eto
Prothionamide	Pto
Cycloserine	Cs
Terizidone	Trd
Para-Aminosalicylic Acid	PAS
Linezolid	Lzd
Clofazimine	Cfz
Amoxicillin and Clavulanic Acid	AmxClv
Imipenem-Cilastatin	Ipm
Meropenem	Mpm
Bedaquiline	Bdq
Delamanid	Dlm
Pretomanid	Pa
Perchlozone	Pcz
Thioacetazone	T
Rifapentine	Rpt
Second Line Injectables	SLI
Fluoroquinolones	FQ

**Drug Name / Drug Class of Antiretroviral Therapy**	**Abbreviation**
Nucleoside/Nucleotide Reverse transcription Inhibitor	NRTI
Abacavir	ABC
Didanosine	ddl
Emtricitabine	FTC
Lamivudine	3TC
Stavudine	d4T
Tenofovir alafenamide	TAF
Tenofovir disoproxil fumarate	TDF
Zidovudine	AZT or ZDV
Non-nucleoside Reverse transcription Inhibitor	NNRTI
Delaviridine	DLV
Efavirenz	EFV
Etavirine	ETR
Nevirapine	NVP
Rilpivirine	RPV
Protease Inhibitor	PI
Amprenavir	AMV
Atazanavir	ATV
Darunavir	DRV
Fosamprenavir	FPV
Indinavir	IDV
Lopinavir + ritonavir	LPV/r
Nelfinavir	NFV
Saquinavir	SQV
Tipranavir	TPV
Fusion Inhibitor	FI
Enfuviritide	ENF or T-20
CCR5 Antagonist	CCR5
Maraviroc	MVC
Integrase Inhibitor	II
Bictegravir	BIC
Dolutegravir	DTG
Elvitegravir	EVG
Raltegravir	RAL

SYSTEM/ORGAN CLASS
Blood and lymphatic system disorders
Cardiac disorders
Congenital, familial and genetic disorders
Ear and labyrinth disorders
Endocrine disorders
Eye disorders
Gastrointestinal disorders
General disorders and administration site conditions
Hepatobiliary disorders
Immune system disorders
Infections and infestations
Injury, poisoning and procedural complications
Investigations
Metabolism and nutrition disorders
Musculoskeletal and connective tissue disorders
Neoplasms benign, malignant and unspecified (incl cysts and polyps)
Nervous system disorders
Pregnancy, puerperium and perinatal conditions
Psychiatric disorders
Renal and urinary disorders
Reproductive system and breast disorders
Respiratory, thoracic and mediastinal disorders
Skin and subcutaneous tissue disorders
Social circumstances
Surgical and medical procedures
Vascular disorders

WHO 2013 Outcome Definitions (Preferred)	
**Outcome**	**Definition**
Cure	Treatment completed as recommended by the national policy without evidence of failure AND three or more consecutive cultures taken at least 30 d apart are negative after the intensive phase (or Month 8 if no intensive phase).
Complete	Treatment completed as recommended by the national policy without evidence of failure BUT no record that three or more consecutive cultures taken at least 30 d apart are negative after the intensive phase (or Month 8 if no intensive phase).
Failure*	Treatment terminated or need for permanent regimen change of at least two anti-TB drugs because of: (1) lack of conversion by the end of the intensive phase, or (2) bacteriological reversion in the continuation phase after conversion to negative, or (3) evidence of additional acquired resistance to fluoroquinolones or second-line injectable drugs, or (4) adverse drug reactions.
Death	A patient who dies for any reason during the course of treatment
Lost to Follow-up	A patient whose treatment was interrupted for 2 consecutive months or more.

WHO 2005 (Laserson) Outcome Definitions (if 2013 not possible)	
**Outcome**	**Definition**
Cure	Completed treatment according to program protocol and has at least five consecutive negative cultures from samples collected at least 30 d apart in the final 12 mo of treatment. If only one positive culture is reported during that time, and there is no concomitant clinical evidence of deterioration, a patient may still be considered cured, provided that this positive culture is followed by a minimum of three consecutive negative cultures taken at least 30 d apart.
Complete	Completed treatment according to program protocol but does not meet the definition for cure because of lack of bacteriological results (i.e., fewer than five cultures were performed in the final 12 mo of treatment).
Failure	Treatment will be considered to have failed if two or more of the five cultures recorded in the final 12 mo of therapy are positive, or if any one of the final three cultures is positive. (Treatment will also be considered to have failed if a clinical decision has been made to terminate treatment early because of poor clinical or radiological response or adverse events).
Death	A patient who dies for any reason during the course of MDR/RR-TB treatment
Lost to Follow-up	A patient whose treatment was interrupted for two or more consecutive months for any reason without medical approval.

Example of an Initial Data Sharing Agreement (Can Be Modified on a Case-By-Case Basis)

LETTER OF AGREEMENT for IPD in MDR/RR TB

This letter of agreement is between the McGill University group (hereafter referred to as the McGill group) for an Individual Patient Data (IPD) meta-analysis in multidrug-resistant tuberculosis TB (MDR-TB), and [INSERT NAME OF INVESTIGATOR AND INSTITUTION] (hereafter referred to as the investigator), regarding the transfer and use of data collected by the investigator. The McGill group and the investigator agree to collaborate on [INSERT NAME OF PROJECT] according to the terms in this letter and those set out in the full project protocol, which is attached as Annex 1.

The McGill group agrees to:

- Obtain approval from the Research Ethics Board of the Montreal Chest Institute, McGill University Health Center for this research.
- Respect the confidentiality of all data received. They will not attempt to identify patients, nor contact patients directly.
- Respect the principle that the investigator continues to 'own' the data sent for inclusion in this analysis. When the data set is "cleaned" and preliminary analyses completed, a copy of the data set will be returned to the investigator.
- Perform data analysis that addresses the objectives specified in the attached study protocol only. Any additional analysis will be performed only after it has been approved by the investigator. For additional analyses that are closely related to these objectives, the investigator will be informed; approval will be assumed if the investigator does not reply within a specified interval. If the investigator has concerns or objections to any new analyses, these will be addressed and resolved before proceeding. Analyses to address completely novel objectives that have not been foreseen in the current study protocol must be actively approved by the investigator before these analyses are undertaken.
- Finish analyses and return the data to the investigator by the sunset date. This date will be the date by which the analyses must be completed, and any manuscript(s)

prepared. The tentative sunset date to complete analyses, and prepare related manuscripts is [INSERT DATE]. If a manuscript is submitted, the data must be held until peer review is completed, and then up to 1 year after publication – to allow time for responses to the findings (e.g., letters to the editor). However, after the sunset date no further new analysis can begin without agreement to the extension of the sunset date by the investigator.

• Share results of analyses with the investigator, and all members of the IPD group at intervals described in the study protocol.

• Prepare draft and final reports of results for the project and prepare manuscript(s) of results for publication. All draft reports and manuscripts will be reviewed and approved by the investigator, and all members of the IPD group before submission. The authorship of these reports will be "The Collaborative Group for Meta-Analysis of Updated Individual Patient Data in MDR-TB", followed by a listing of all members – in alphabetic order. The corresponding author will be Dr. Menzies of McGill.

The investigator agrees to:

• Verify whether they require approval from their local Research Ethics Board, depending on their institution's policy. If so, the investigator will obtain this approval before sending the data to the McGill group. No additional data will be collected from the patients, thus investigator will not need to obtain patients' consent for this analysis.

• Transfer a data file of information on all patients who were members of a cohort of MDR-TB patients which the investigator reported in earlier publications. This patient dataset will be rendered completely anonymous before forwarding this to the Montreal Chest Institute by removing all personal identifiers.

• Become a member of The Collaborative Group for Meta-Analysis of Updated Individual Patient Data in MDR-TB. This Collaborative Group will review all preliminary and final results of analyses performed by the McGill group, as well

as all reports of results – for the guideline groups, for public presentation, and for publication.

- Treat these preliminary results confidentially. The investigator will not publish (including posting on the Internet), present in any public forum, nor disseminate through any media these results without approval from the McGill Group and other members of the IPD Collaborative Group.

____ __________________

Dr. Dick Menzies (for the McGill University Group)   Date

____ __________________

[Insert name and institution]   Date
